# Physical and social environmental changes to promote walking among Dutch older adults in deprived neighbourhoods: the NEW.ROADS study

**DOI:** 10.1186/s12889-016-3563-2

**Published:** 2016-08-31

**Authors:** R. G. Prins, C. B. M. Kamphuis, J. M. de Graaf, A. Oenema, F. J. van Lenthe

**Affiliations:** 1 Department of Public Health, Erasmus University Medical Center Rotterdam, Rotterdam, The Netherlands; 2 MRC Epidemiology Unit and UKCRC Centre for Diet and Activity Research (CEDAR), School of Clinical Medicine, University of Cambridge, Cambridge, UK; 3 Department of Human Geography and Spatial Planning, Faculty of Geoscience, Utrecht University, Utrecht, The Netherlands; 4 Department of Health Promotion, Maastricht University, Maastricht, The Netherlands

## Abstract

**Background:**

Physical activity is important for healthy ageing, and daily walking is seen as a feasible way to be active at older ages. Yet, many older persons, particularly in lower socioeconomic groups and residing in deprived neighbourhoods, are insufficiently active. Creating a physical and social neighbourhood environment that is more supportive for walking has the potential to improve walking behaviour. Current evidence of the impact of changes to the physical and/or social environmental on walking behaviour is scarce. The aim of the NEW.ROADS study is to design, implement and evaluate changes to the physical and social environment for the purpose of increasing walking behaviour among older residents of deprived neighbourhoods.

**Methods:**

Physical and social environmental interventions were developed by matching scientific evidence on environmental determinants of walking, with input from the target population and stakeholders, and ongoing neighbourhood activities. Specifically, a neighbourhood walking route was designed and marked, and neighbourhood walking groups were organised. These environmental interventions were evaluated in a four-armed experimental study. In addition, the design of the study to evaluate the effect of these environmental changes on walking behaviour is described.

**Discussion:**

Designing and implementing environmental interventions is a complex endeavour, challenged by limited available theory and evidence. Input from the target population and professional stakeholders is essential, but may also put constraints on the evaluation.

**Trial registration:**

NTR3800 (registered 9/1/2013)

## Background

Physical activity is a crucial element of healthy ageing [1–3], as it may prevent major non-communicable diseases, including coronary heart diseases, type-2 diabetes, cancer [4], falls [5], and depression [6], and may increase mobility [7]. Evidence suggests that even moderate physical activity may reduce the risk of premature mortality among older persons [8]. Yet, one out of three adults is insufficiently physically active worldwide [9].

Levels of physical activity are unevenly distributed according to socioeconomic position (SEP). The inverse association between individual SEP and leisure time physical activity has been extensively described [10–13]. Further, ample studies reported older residents of deprived neighborhoods to be more often inactive as compared to those residing in more affluent neighborhoods [14–16]. Thus, there is a need to promote physical activity among a wide population of older adults, and particularly among the socioeconomically disadvantaged. Walking is seen as important type of physical activity for this purpose; it does not require specific facilities or equipment, can be done at older ages and for free.

Improvements in physical activity levels are most likely when interventions and policies are targeted to the most salient determinants of physical activity. Socio-ecological models articulate that both individual and environmental factors influence physical activity [17]. Environmental factors may be particularly relevant for older persons, who are, due to their lower mobility, more likely to be dependent on their immediate living environment. Previous research on environmental determinants of physical activity has to a large extent focused on physical environmental factors, and has most consistently found that highly walkable neighborhoods are related to more walking among older persons [18]. Other studies found relationships with green [19], functional design and levels of safety [20, 21]. Although less often studied, evidence also suggests an important role for social environmental factors, perhaps most notably social support [22, 23].

Socio-ecological models suggest that interventions are likely to have the greatest effect when determinants at different levels are addressed simultaneously [24–26]. However, surprisingly little is known about the combined role of physical and social environmental characteristics on physical activity. Recent observational research confirmed however, that the availability of trails had a stronger influence on walking when walking partners were around [27]. Further, the availability of parks was more strongly associated with sports participation with increasing levels of neighbourhood social capital among youth [13]. While the limitations of observational evidence with regard to causal inference are generally recognized, the number of studies in which changes to the environment have been evaluated is still small [28]. For the ultimate purpose of creating walkable neighborhoods, more insight in causality is pivotal. Therefore, in the NEW.ROADS study, we aim to develop, implement and evaluate social and physical environmental interventions to promote walking behaviour among older adults living in deprived neighbourhoods. The current paper describes the development of these interventions and the design of the evaluation study, in which the separate and combined effects of the social and physical environmental interventions were tested.

## Methods

### Setting

The study is situated in Rotterdam, which is the second largest city of the Netherlands. Compared to the rest of the Netherlands, Rotterdam is a city with a relatively high proportion of deprived neighbourhoods. Four of these deprived neighbourhoods (Bloemhof, Hillesluis, Nieuwe-Westen, and Tarwewijk) were selected because of their reasonable similarity in socioeconomic composition and physical infrastructure.

### Development of the environmental interventions

The development of the physical and social environmental intervention was based on three elements: 1) scientific evidence on important environmental determinants of walking among older persons, 2) input from the target population about environmental factors perceived to be relevant for walking and 3) the need to align the interventions with ongoing activities in the neighbourhoods as organised by other stakeholders.

#### Scientific evidence on environmental determinants of walking among older adults

A systematic review on physical environmental correlates of physical activity in older adults found few consistent results [29]. However, some evidence suggested that land-use mix diversity was associated with walking. Also, associations were reported between access to shops and recreational walking, and between neighbourhood walkability and walking for transportation purposes. Given that salient environmental determinants may depend on the national context [30], we gave more importance to study findings obtained in the Dutch context. These studies reported a positive association of the availability of destinations and aesthetics with walking in older adults [21, 31]. Routes through parks were found to be a barrier for physically activity among Dutch older adults [32].

With regard to social environmental characteristics, more consistent results have been reported. Seeing others being active [33, 34], experiencing high levels of social cohesion [35, 36] and peer-support [37, 38] were positively associated with walking behaviour among older adults. A Dutch study reported an association between larger social networks and walking among older adults [31].

#### Needs assessment of the target group: focus group studies with older adults

A well-described challenge when investigating environmental determinants of physical activity, is that objectively measured environmental characteristics do not need to match with how these characteristics are perceived by residents [39], and this may vary by socioeconomic group [40]. For instance, the score for availability of green spaces may be high based on GIS-data, but the accessibility of these green spaces may be perceived as poor by residents (e.g. since they feel unsafe). To get more insight in which physical and social environmental factors are important for walking as perceived by the target group, we conducted four focus group interviews with 5-12 older adults from deprived neighbourhoods in which the environmental interventions would be implemented.

In the focus group interviews, older adults indicated, that the availability of shops and benches are important for walking. They also emphasized the importance of aesthetically pleasing features, such as green, water and seeing the ducks swimming in the pond. When walking alone, participants mentioned to avoid walking along dense bushes, as this was perceived as unsafe.

With regard to the social environment, focus group participants indicated their neighbourhoods as lively, which both had a negative and positive connotation. Positive elements of the neighbourhoods included the high level of social contacts, young children playing on the streets and big markets attracting many clients. Negative aspects of the liveliness however, were burglary, robbery, noise, disrespectful youth and drunks. Participants described how they were sometimes afraid of being ripped and harassed when walking through the neighbourhood, However, they also indicated that walking together may mitigate some of their safety concerns.

The focus groups further showed that older adults liked outings, preferably when these included having a cup of coffee or tea together. Older adults indicated that peer support was an important factor for walking. In addition, seeing others being active was important for some older adults.

#### Collaboration with stakeholders on planned or ongoing activities

Developing and implementing an environmental intervention requires collaboration with stakeholders [41], for instance to fine tune the interventions with plans by local governments, and to generate support of community organizations. In the neighbourhoods that were selected for the interventions, a variety of municipal programs and grassroots organisations already organised initiatives aimed at improving health and wellbeing. We familiarised ourselves with these initiatives, and tried to collaborate with the initiatives that fitted well with our aims. In fact, we decided to form a multi-disciplinary project team. This enabled us to identify projects with goals similar to those of our interventions, such as a marked walking route planned in one of the neighborhoods and a programme aimed at training lay-man to help people getting more active (the Physical Activity Buddy program). Such collaborations meant that the interventions partly built upon ongoing initiatives, which were not necessarily theory- and evidence-based. This constrained the development of the interventions to some extent (e.g. route markers for walking routes were already chosen), however, the alignment with stakeholders and ongoing activities was seen as an important advantage for a successful implementation of the interventions.

The combination of available scientific evidence, input from the target groups and the wish to align and collaborate with initiatives from other stakeholders resulted in an intervention in which the physical component consisted of the design of a walking route, and the social component consisted of the initiation of lay-men organised walking groups.

### Implementation of the physical environmental change: design of a neighbourhood walking route

**Fig. 1:**
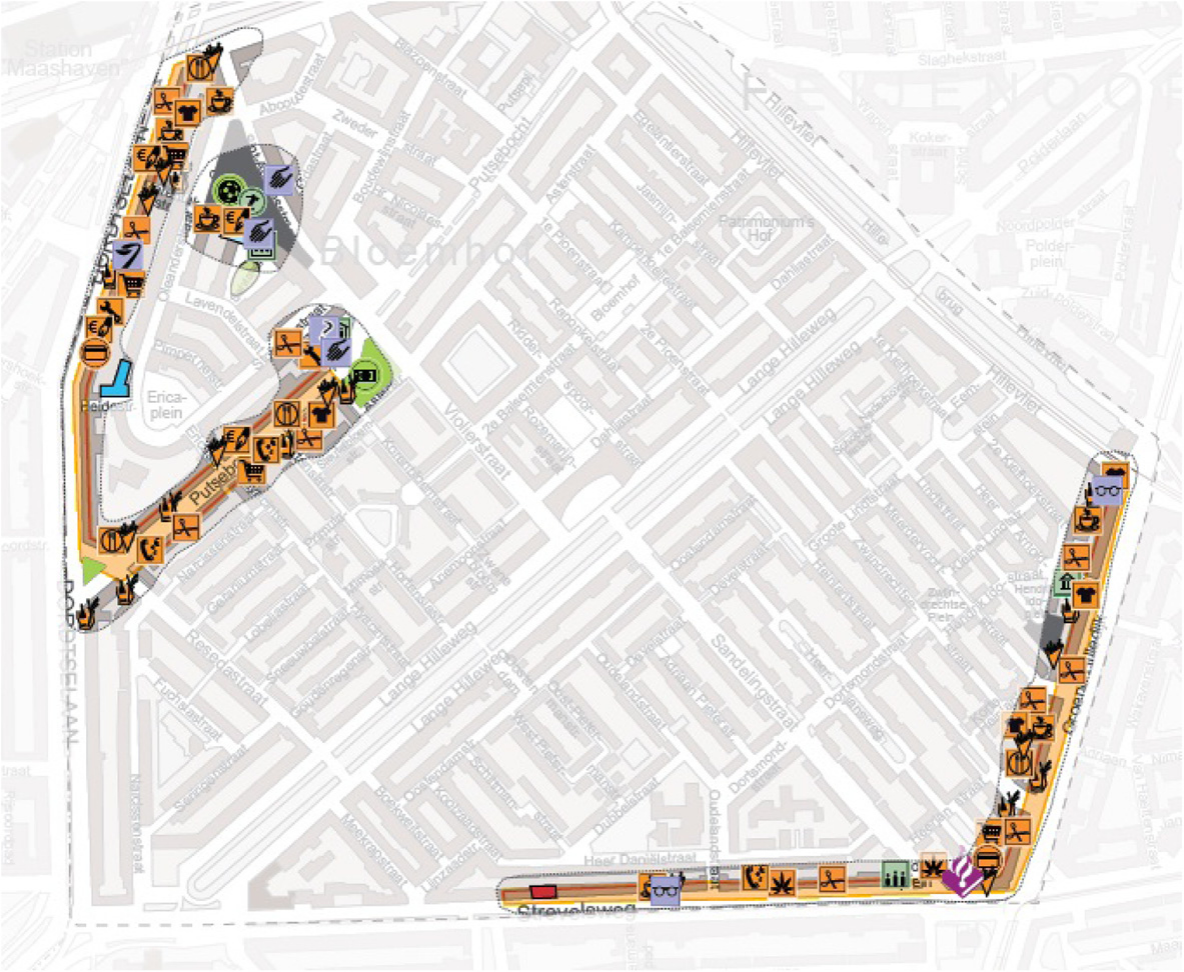
GIS-based anchor point analysis of relevant hotspots in one the neighbourhoods

The first step in determining the walking route was the visualisation of the availability of important destinations such as shops and GPs in each neighbourhood. Municipal and commercial databases were used with information on the availability of shops and relevant facilities for older adults, and these were put on a GIS-based map (Fig. 1). This was done by a knowledge institute (“De Veldacademie”) with whom we collaborated. The areas with the highest density of these facilities were treated as hotspots (“anchor points”), thereby emphasizing the importance of their connectivity and interrelatedness.

Once the important items on “accessibility” were mapped, the maps were enriched with information derived from the focus groups and field observations to capture safety related elements. For example, focus group participants sometimes explicitly stated that they perceived some areas as dangerous and others as very attractive to walk. These maps were discussed with municipal policy makers, urban planners and welfare workers that participated in the project team. Based on their local knowledge, elements of “pleasurability” were added to the map (e.g. street art exhibitions, monumental houses or streets, or historical sites) and less attractive parts were removed. The locations that were mapped formed the basis of a first draft of the routes.

The routes were then walked by two researchers (RP, JdG) to identify issues not yet mapped, such as the availability of benches, presence of litter and graffiti, signs of vandalism. In addition we used the observations to check for other important factors mentioned in the Hierarchy of Walking needs such as the quality of the pavement, completeness of the pedestrian network and accessibility of the route with walking frame or wheelchair [42]. The routes were modified accordingly. Finally, the principal investigator (RP) walked the definitive routes with a policy advisor and an urban planner of the municipality, to check whether the route could be implemented and to get approval for construction of the route.

#### Route markings

The idea of the grass root organization with which we collaborated was to add art to the neighborhood, thereby making the neighborhood more colorful. Children of local primary schools could enter a competition by designing a 30 by 30 centimeter paving stone and by submitting the design (on paper) to the organization. The best designs were selected for a workshop in which the paving stones were painted. This process had already started before we initiated our collaboration. Together with the grass roots organization we extended this project within the neighborhood in which they started (Bloemhof) and extended this to other neighborhoods in which we planned a walking route.

#### Implementing the route

On each corner two painted paving stones were placed in such a way that the direction of the route was logical from both directions. In addition, additional painted paving stones were placed on segments that consisted of long straight streets, to confirm walkers that they were still on the right track.

#### Promotion of the routes

The walking routes were promoted via various channels: local media campaigns (initiated by the grassroots initiative), an opening event of the route (by an alderman) and a door-to-door spread of a glossy route guide and map within the neighbourhood in which the route was created.

### The implementation of the walking groups

Based on the needs of the target population and previous evidence for their effectiveness on an individual level [43, 44], we decided that organising walking groups would be the social intervention. The older adults in our focus groups indicated that seeing other people walk improved the likelihood that they would go for a walk. Therefore, the walking groups were specifically meant to walk through their neighbourhood, so that this may affect people not participating in the walking groups as well. We collaborated with the Municipal Health Services Rotterdam and a non-governmental organisation (NGO, named Avant Sanare) and developed the intervention by using their Physical Activity Buddies System (PABS). The PABS is an extension of the Beweegkuur [45] and aims to target hard-to-reach groups and stimulate them to be more physically active. In PABS, those who experience a barrier to be physically active (“searchers”) are matched with experienced exercisers (“PA buddies”).

The PA buddies received an extensive training, led by a qualified educationist, on motivating people to be physically active and maintaining this motivation. After the training the PA buddies could either be matched with “searchers” through a database maintained by the Municipal Health Services, or by setting up their own groups via their own networks. Moreover, regular peer support meetings and supervision meetings were planned throughout the year. The approach of PABS fits well with the Volunteer Lay Leader Model – in which volunteers with a similar background as the target group has proven successful in interventions targeted at hard-to-reach groups. This model has proven effects on disseminating falls prevention program among older adults [46] and improvement of self-management among diabetics [47]. The PABS program was however not specifically aimed at groups of older adults.

#### Adapting the physical activity buddies system to facilitate walking groups

PABS provided us with a good infrastructure to train interested (active) people from the general public to become walking group leaders and start walking groups. Based on the course materials and manual, an additional course session was written aimed at teaching how to set-up a walking group and deal with functional limitations of older adults.

In all focus group interviews we conducted, drinking coffee or tea afterwards was mentioned as a big incentive for older adults to take part in an activity. This was also recognized by the experts in the study team. Therefore it was decided that a component of the walking groups was a joint closure with coffee and tea. This was subsidized by the study for the first 12 sessions.

#### Promotion of the walking groups

The walking group leaders were instructed to attract people from their own neighbourhood. Recruitment through word of mouth can be a successful strategy for participation among older adults [48]. Participants and group leaders (PA buddies) were recruited by adverts placed on the front page of local door-to-door newspapers. Furthermore, a coloury flyer was spread door-to-door in the neighbourhoods in which the social intervention was planned to recruit participants and group leaders. Recruitment also took place by using the existing channels of the PABS program.

## Evaluation study

### Aim of the evaluation study

We will study whether changes in the physical and social environment can increase walking among Dutch older adults living in deprived neighbourhoods. Further, we will explore how and for whom these environmental changes affected walking, by applying quantitative and qualitative methods.

### Evaluation design, participants and recruitment

The environmental interventions are evaluated in a four-armed experimental study on changes in minutes walked. These four arms correspond to four neighbourhoods, namely: 1) a neighbourhood with the physical environmental intervention, 2) a neighbourhood with the social environmental intervention, 3) a neighbourhood with both physical and social environmental interventions and 4) a control neighbourhood (no interventions). These neighbourhoods were selected in close collaboration with policy makers on their comparability (demographics, physical environment). Although random allocation to study arms was envisaged, due to collaboration with ongoing activities assignment to the study arms was mostly based on practical reasons (e.g. the walking route project with which we collaborated started in one of the neighbourhoods; which became the physical environmental condition). Measurements will take place at baseline and 3 and 6 months after implementation of the environmental changes.

G*Power was used to perform a sample size calculation [49]. Because the aim of the intervention was to increase walking, our sample size calculations was based on the differences in the change in minutes walked, We used a repeated measures ANOVA design for this purpose, with four groups measured three times. We assumed a small effect size (f) of 0.1. In repeated measures, the correlation between the repeated measurements is also of importance, which we assumed to be 0.5, based on the reliability of the IPAQ (the instrument which was used to measure walking) [50]. Considering a power of 0.8 and a *p*-value of 0.05, a total sample size of 328 older adults is needed [49].

Other studies that recruited people from a “community dwelling” population found responses of 15-17 % [51, 52]. Hence, approximately 700 older adults in each neighbourhood needed to be approached. A sample of 700 older adults aged 55 years and above residing in the selected four neighbourhoods was randomly drawn from the Municipal Inhabitant Registration. In line with recommendations to maximise response to postal surveys, older adults were approached by first sending an invitation letter with a brochure on the study [53]. Two weeks after sending the introduction letter, all older adults who had not objected to take part in the study, received a first questionnaire pack, including an introduction letter, a brochure about the purpose and set-up of the study, the questionnaire, a consent form, and a free-post return envelope. Three to four weeks later, non-responders received another questionnaire pack and were called or visited. To serve as a “thank you” and to boost participation, the introduction letters mentioned the chance of winning gift vouchers of 10 Euro.

A random subsample from the responders to the postal survey was recruited for wearing an accelerometer and a GPS logger for 7 consecutive days during each wave of data collection. The Medical Ethics Committee of the Erasmus University Medical Centre approved this study.

### Conceptual framework

**Fig. 2:**
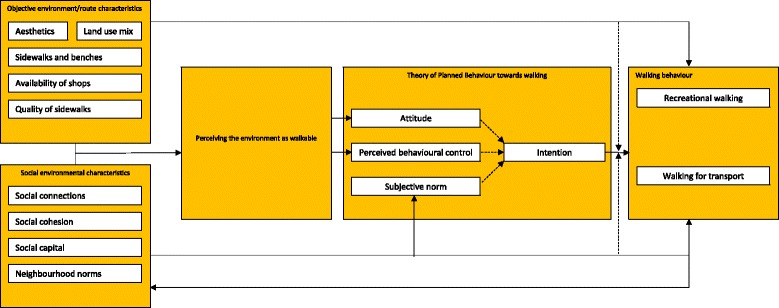
Conceptual framework of the NEW.ROADS study

Empirical evidence on mechanisms by which environmental factors influence physical activity is largely lacking [54]. Therefore, we drew on socio-ecological frameworks and observational studies to conceptualise how physical and social environmental factors may influence walking behaviour among older adults. Our conceptual framework is depicted in Fig. 2, and suggests a direct influence of the environment on walking behaviour [55] and two indirect mechanisms:

1. Mediation by perceived environmental and cognitive factors: Environments conducive to walking are hypothesized to positively influence perceptions of the walking environment [42, 55], which may positively influence motivational determinants of walking (Fig. 2), such as attitudes towards walking.
2. Moderation of the relation between the intention to walk and walking behaviour. Social and physical environmental factors may facilitate transferring intentions to behaviour [56–58]. In other words, environmental changes may help to mitigate the intention-behaviour gap (i.e. no behaviour change despite having a positive intention towards the new behaviour) which was found for different lifestyle behaviours [59], including physical activity [60, 61]. Thus, for older adults with positive intentions to walk, modifying the physical and social environment may facilitate them to act accordingly (Fig. 2).

### Measurements

For the overall evaluation of the interventions in terms of walking behaviour, and to understand the underlying mechanisms, participants receive a questionnaire pack at each time point. Table 1 schematically shows which constructs will be measured at each time point.

**Table 1:** Timeline of measurements during the effect and process evaluation of the NEW.ROADS study

	*T0*	*T1*	*T2*
Time	February/March	August/September	November/December
Primary outcomes	*Self reported walking (IPAQ)*	*Self reported walking (IPAQ)*	*Self reported walking (IPAQ)*
Secondary outcomes	*MVPA (IPAQ)* *Perceived health (SF12)*	*MVPA (IPAQ)*	*MVPA (IPAQ)* *Perceived health (SF12)*
Determinants	*Perceived environmental factors (NEWS)* *Social cohesion and social capital* *Theory of Planned Behaviour constructs*	*Perceived environmental factors (NEWS)* *Social cohesion and social capital* *Theory of Planned Behaviour constructs*	*Perceived environmental factors (NEWS)* *Social cohesion and social capital* *Theory of Planned Behaviour constructs*
Process evaluation	*GPS*	*GPS* *Walk along interviews*	*GPS* *Questionnaire: awareness, use and appreciation* *Phone interviews*

#### Outcomes

Self-reported physical activity will be measured by means of the IPAQ long version [62]. The primary outcome measure will be weekly minutes spend walking in leisure time and for transport. Secondary outcome measures include moderate-to-vigorous physical activity (IPAQ), objectively measured levels of MVPA (measured with accelerometers) and perceived health [63].

#### Understanding the underlying mechanisms: mediating factors

To study the proposed mechanisms, we will measure neighbourhood perceptions, using the Neighbourhood Environment Walkability Scale (NEWS) [64], Theory of Planned Behaviour constructs [65] and social cohesion and social capital constructs in questionnaires.

#### Understanding the intervention effect: process evaluation

The process evaluation of NEW.ROADS will consist of various components with the aim to understand the awareness, use acceptability, feasibility, implementation and appreciation of the interventions among participants. This will be done by using a number of activities ranging from the analysis of GPS tracks and accelerometer data, to conducting walk-along interviews, and analysis of questionnaire data.

Whether people use the walking routes will be measured in a 10 % subsample of older adults, by means of GPS loggers (Travel recorder X, BT-Q1000X, QStarz International). The BT-QStarz GPS logger has a relatively good performance in terms of accuracy and battery life. The participants will be instructed to charge the GPS logger overnight, while keeping it switched on – previous studies have shown high compliance with this procedure (Jones et al., [66]).

To get insight in the perceptions of the participants on the walking route, walk-along interviews will be held with 30 participants from neighbourhoods in the physical environment condition. During these walk along interviews, the participants walk a part of the walking route with members of the research team. The walk will be recorded with two voice recorders (one for the member of the research team and one for the participant) and a GPS logger. Moreover, geo-coded photographs will be taken from important physical or social environmental characteristics.

In the final questionnaire at T2, items about awareness, use and appreciation of the environmental changes from the participant’s point of view will be measured. To get further insight in this, a random selection of 100 participants will be phoned and asked about their awareness of the route and ways to further improve the route.

## Discussion

In this paper we described the development and evaluation protocol of physical and social environmental interventions to promote walking behaviour among older adults living in deprived neighbourhoods. It shows that developing, implementing and evaluating such environmental interventions is challenging, given specific circumstances in the local context in which these interventions were implemented (i.e. specific wishes from the target group, and ongoing activities of other stakeholders).

First of all, the theory and evidence base for developing interventions that modify the physical environment in order to promote physical activity is small. While some socio-ecological frameworks are available, only some of them explicitly inform which environmental factors specifically impact upon physical activity, and what the underlying mechanisms are. Calls have been made to come up with a better understanding of how, when and for whom environmental interventions work [54]. We acknowledge that such understanding is not yet as elaborate as seen in the field of health psychology [57, 67].

A second challenge is that the interventions described in this paper are implemented in ‘the real’ world, which is out of control of the researcher [68]. This means, for instance, that other initiatives may be ongoing with similar goals as the interventions to be developed. In this project, cooperation with various organisations helped us to identify other parties that were already operating (or starting to operate) in the field and strengthen each other’s projects. Therewith we experienced ourselves how important cooperation with local organisations and with different disciplines is. On the other hand, it should be noted that these collaborations also constrain the development and evaluation of interventions for researchers. In our case the decision of the specific route markers in the physical environmental intervention (i.e. painted tiles) was already made, whereas other route markers) could have had advantages over tiles, for instance with regard to visibility. However, ignoring the potential for cooperation would increase the costs, which is undesirable from a cost perspective. An associated challenge is that intervention implementation is out of control of the researcher. Whereas the walking routes were implemented in close collaboration with the project team, by nature, the social environmental interventions were under less control of the research team.

To conclude, we described the development and evaluation of two interventions, one targeting the social neighbourhood environment and the other targeting the physical neighbourhood environment. The evaluation study will provide evidence for their separate and combined effects on walking behaviour of residents. Moreover, it will shed light in how changes to the environment may cause changes in walking behaviour.

## Abbreviations

GIS: Geographic information system
GP: General practitioner
GPS: Global positioning system
MVPA: Moderate-to-vigorous physical activity
NEWS: Neighbourhood environment walkability scale
NGO: Non-governmental organisation
PA: Physical activity
PABS: Physical activity buddy system
SEP: Socioeconomic position
